# Artificial Intelligence-Enabled 8-Channel ECG Diagnosing of Abnormalities with Wide QRS Complexes

**DOI:** 10.34133/hds.0265

**Published:** 2026-02-05

**Authors:** Hongling Zhu, Qiushi Luo, Yao Wang, Mairihaba Maimaiti, Heng Zhang, Yulong Xiong, Chen Ruan, Jingyi Wang, Yedan Liu, Mengqiao Zhou, Yinan Sun, Wei Chen, E. Jin, Jin Li, Xia Chen, Tao Zhu, Xiaoyun Yang

**Affiliations:** ^1^ Division of Cardiology, Department of Internal Medicine, Tongji Hospital, Tongji Medical College, Huazhong University of Science and Technology, Wuhan, Hubei 430030, China.; ^2^ Wuhan Zoncare Bio-medical Electronics Co. Ltd., Wuhan, Hubei 430206, China.; ^**3**^ Department of Cardiac Function, Jingmen Central Hospital affiliated to Jingchu University of Technology, Hubei 44800, China.; ^4^ Department of Computer Center, Tongji Hospital, Tongji Medical College, Huazhong University of Science and Technology, Wuhan, Hubei 430030, China.; ^5^ Cardiac Function Department, Department of Internal Medicine, The First People’s Hospital of Jiangxia District, Wuhan, Hubei 430200, China.

## Abstract

**Background:** There is a substantial number of research exploring the application of artificial intelligence (AI) in identifying electrocardiogram (ECG) abnormalities related to heart rhythm or conduction with the 12-channel format. However, there is a scarcity of studies focusing on refined differentiation of serials of ECG abnormalities with wide QRS complexes in a simplified channel format. **Methods:** We constructed an ECG dataset (standard 10-s, 12-channel format) from adult patients from Tongji Hospital of Huazhong University of Science and Technology, Wuhan, China. This dataset was consisted of 5 kinds of ECG abnormalities with wide QRS complexes in the normal heartbeat (60 to 100 beats per minute) and the normal ECGs. Convolutional neural network was developed to classify these abnormalities. Four-channel (I, II, V1, and V5) and 8-channel (I, II, and V1 to V6) formats, compared to the standard 12-channel format (I, II, III, aVR, aVL, aVF, and V_1_ to V_6_), were chosen as the input channel format of the model. Other unreplicated ECGs from Tongji Hospital (TJ-Test set), annotated by a committee of board-certified cardiologists, served as the test dataset. The F1 score, area under the receiver operating characteristic curve (AUROC), and accuracy were calculated to assess the performance of the model, which were further compared with diagnoses of 6 ECG cardiologists who were informed that the final objective was classifying among 6 classes with the 12-channel format. In addition, a dataset of 291 ECGs from The First People’s Hospital of Jiangxia District (JX-Test set) and a public dataset of 64 ECGs were used to assess model generalizability **Results:** The dataset consisted of 11,808 ECGs from 8,542 patients from 2012 January 1 to 2020 November 30 and divided into training and validation datasets in the ratio of 9:1. The test dataset included unreplicated 480 ECGs from 480 new adult patients recorded from 2014 January 1 to 2017 November 30. The model shows a superior performance in the 8-channel format compared to that of 4- and 12-channel formats. As for the 8-channel format, the model obtained an accuracy of 95.0%, a mean F1 score of 0.969 (0.943 to 0.997), and a mean AUROC score of 0.997 (0.975 to 1.00) compared to an accuracy of 89.9%, an F1 score of 0.898 (0.863 to 0.932), and an AUROC score of 0.941 (0.918 to 0.963) of physicians assessing the same datasets. The model exhibited a mean F1 score of 0.917 (0.943 to 0.997) and a mean AUROC score of 0.994 (0.975 to 1.00) on the JX-Test set, and mean F1 scores of 0.708 for the left bundle branch block and 0.828 for the right bundle branch block for the external published validation data both with the 8-channel format. **Conclusion:** Our model distinguishes a range of distinct abnormalities focusing on abnormal morphology on QRS complexes in normal heartbeats with high accuracy, providing a foundation for AI-aided clinical decision-support systems in ECG differential diagnosis.

## Introduction

The QRS complex in electrocardiogram (ECG) stands for the depolarization of the heart ventricular and is a crucial component of cardiac electrical activity. It is closely related to the incidence of heart failure, sudden cardiac death, and structural heart diseases. Correct diagnoses of ECG abnormalities exhibiting atypical morphology on the QRS complex are essential for appropriate treatment, while incorrectly interpreted ECGs can lead to adverse outcomes [1,2].

**Fig. 1.**
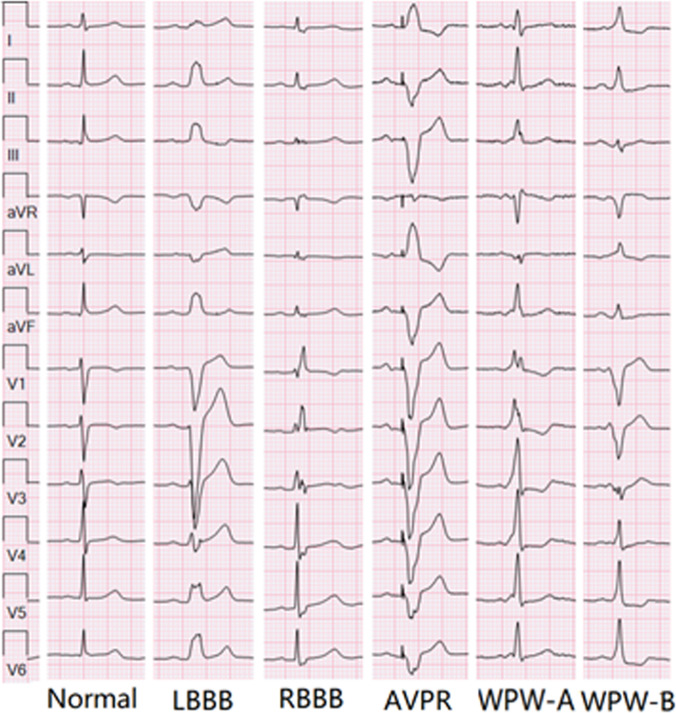
Representative ECG images of the 6 rhythm classes.

However, it is a notable challenge for physicians to diagnose ECGs characterized by abnormal morphology on the QRS complex [3], as it includes various types of ECG conduction abnormalities such as bundle branch block (BBB), artificial ventricular pacing rhythm (AVPR), and pre-excitation syndrome (WPW) [4]. In the type A WPW (WPW-A), an anomalous AV (atrium and ventricle) connection exists between the left atrium and left ventricle, which leads to pre-excitation of left ventricle and vectors toward precordial channels. Therefore, WPW-A exhibits positive delta waves and QRS waves in all precordial channels. These features are often confused with right BBB (RBBB). Likewise, in type B WPW (WPW-B), an anomalous AV connection between the right atrium and right ventricle leads to right ventricular pre-excitation. As a result, WPW-B displays negative QRS waves in the V1 channel and positive delta and QRS waves in V4 to V6, which is often misdiagnosed as left BBB (LBBB). AVPR also shows similar wide waveforms as LBBB, which again increases the difficulties in diagnosing the above ECG abnormalities (Fig. 1).

Currently, AI-enabled ECG diagnosing has been widely studied on ECG abnormalities with abnormal morphology on the QRS complex [5,6]. Zhu et al. [7] developed a deep learning model to classify 21 arrhythmias of heart rhythm and conduction abnormalities using 12-channel ECGs. The model shows promising F1 scores on both internal and external database, even higher than that of experienced cardiologists. Makimoto et al. [8] developed a convolutional neural network (CNN) to recognize ECGs of myocardial infarction (MI), and the model shows a higher F1 and accuracy than those of the physicians. Senoner et al. [9] identified the location of an abnormal pathway in WPWs with an neural network (locAP AI) in 357 consecutive WPW syndrome patients using 12-channel ECGs, and the model obtained an accuracy of 85.7%. However, there are seldom studies focusing on refined differential diagnosis of serials of ECG abnormalities with wide QRS complexes.

What is more, the diagnosing guidelines for the above ECG abnormalities related with all 12-channel ECG and the algorithms are commonly constructed to be accustomed to 12-channel ECG forms [10,11]. This would support the model with comprehensive information on the ECG changes, but on the other hand, it obviously increased the time for data processing and data application and the room for data storage. Thus, limited channel formats have been studied. Luo et al. [12] constructed a CNN model in identifying atrial septal defect among adults using 8-channel ECG, and the model shows an F1 score of 0.81 and an AUC (area under the receiver operating characteristic curve) of 0.87. Different kinds of channel formats remain unexplored yet in ECG abnormalities with wide QRS complexes.

Consequently, we addressed the challenge in classifying ECG abnormalities with a wide QRS complex with sinus rhythm with heartbeat between 60 and 100 beats per minute, which stand for complex and confusing abnormalities that need different treatment, and are reported frequently in the clinical practice. We excluded the wide QRS tachycardia (WCT) that manifests differently and depends on different crucial interventions compared to normal rates. We developed a general AI-based ECG diagnosis model that covers a series of similar waveforms and tried the different channel format in AI-based diagnosing.

## Methods

### Data sources and study population

Our study was carried out in accordance with relevant regulations and guidelines [5,7]. As described previously [7], the ECG dataset was consisted of retrospective data from adult patients (≥18 years) who had an ECG at Tongji Hospital of Huazhong University of Science and Technology, Wuhan, China, from all 3 campuses. The ECGs were recorded at standard 10-s, 12-channel format with a sampling rate of 500 Hz, mainly using the GE-Marquette ECG machine (GE Healthcare, Milwaukee, WI, USA), Zoncare ECG machine (Zoncare Electronics Co. Ltd., Wuhan, China), and 24-h dynamic ECG machine (DMS Holter Company, Stateline, NV, USA). The MUSE data management system (GE Healthcare) was used to store the raw data. The ECGs were labeled into 6 specific classes, including LBBB, RBBB, AVPR, WPW-A, WPW-B, and the normal group. The dataset was further divided into training and validation datasets without any patient overlap in a ratio of 9:1. We collected a set of ECGs from Tongji hospitals as a test dataset corresponding to unique patients not included in the training and validation dataset.

An open access dataset containing 827 ECGs publicly available on the link, https://zenodo.org/record/3765780#.YVIM8J5Kgl9%2F, was used for external validation to examine the generalizability of the proposed approach. This external dataset contained about 6 different abnormalities, in which we selected 2 of them of the same abnormalities including RBBB and LBBB as found in our dataset to use for the external validation [13].

We utilized data that had been anonymized to guarantee patient confidentiality. The team in charge of data collection was responsible for gathering samples and ensuring their anonymization. Meanwhile, the algorithm team received anonymized data limited to the patient’s identification number, age, and gender for subsequent algorithm development. This study did not necessitate written informed consent, as the ECG samples were appropriately anonymized and deidentified in accordance with the Health Insurance Portability and Accountability Act Safe Harbor provision [14]. This study was approved by the ethics committee of Tongji Hospital of Huazhong University of Science and Technology (approval number TJ-IRB202507044).

### Annotation procedure

As described previously [7], to guarantee the accuracy of the baseline diagnostic labels, ECGs from the training, validation, and test datasets were initially diagnosed by a primary cardiologist. These ECGs were then subject to further reviewed by senior board-certified cardiologists in the Cardiac Function Examination Center of Tongji Hospital. To ensure the consistent format of data input, the ECGs from the Holter machines was segmented to 10 s. Labels of the 10-s 12-channel ECGs were in agreement with the clinical diagnoses made by cardiologists.

### Physician evaluation

To help assess the comparative performance of the model, we invited 5 ECG physicians from Tongji Hospital, Wuhan, China, and one ECG physician from The First People’s Hospital of Jingmen, Jingmen, China to this study. They are working in cardiology departments, and they are not responsible for the original ECG annotations in the dataset. Every participating physician received specific instructions on how to annotate ECGs in the testing dataset to ensure consistency.

### Overview of the artificial intelligence model

Our model is constructed based on Resnet, which is a network combining the advantages of CNN and solving the problem of gradient vanishing as the network layer deepens. It reduced the optimization difficulties by introducing shortcut connections, which can deepen the number of layers and also have a better network learning effect. Our network structure consists of 37 layers, and there are 15 basic blocks in the middle of the network structure. Each block is composed of 2 convolutional layers connected by shortcut, and the entire network can still be trained through end-to-end backpropagation. Moreover, each convolutional layer is preceded by batch normalization (BN) and ReLU. BN helped improve the model’s stability during training by normalizing the inputs to each layer. The ReLU introduced nonlinearity to the model, enabling it to learn complex patterns. Besides, the max pooling layer helps reduce the computational load by compressing the feature map processed by previous layers. The global average pooling layer generates the final features and reduces the risk of overfitting by largely decreasing the number of parameters. The last layer consists of a softmax layer, which computes the posterior probability of each class through a softmax function.

Four-channel (I, II, V1, and V5) and 8-channel (I, II, and V1 to V6) formats, compared to the standard 12-channel format (I, II, III, aVR, aVL, aVF, and V_1_ to V_6_), were chosen as the input channel format of the model. We used 8 channels (I, II, and V1 to V6) instead of the full 12-channel ECG for 2 reasons. First, channels III, aVR, aVL, and aVF can be calculated from channels I and II, so no key information is lost [15]. Second, V1 to V6 cover important chest areas and are highly sensitive to wide QRS abnormalities, which helps ensure diagnostic accuracy. Regarding the 8-channel format, channels III, aVR, aVL, and aVF are deleted in the standard 12-channel format, because they can be expressed by channels I and II. The mapping formula is defined as follows: III = II − I; aVR = −0.5*(I + II); aVL = I − 0.5*II; aVF = II − 0.5*I. The length of the signal is 10 s, with a sampling frequency of 500 Hz, resulting in an input dimension of the model that is 4 × 5,000, 8 × 5,000, or 12 × 5,000 (depending on the configuration). The model outputs 6 values, each representing the probability that the signal belongs to one of the 6 categories. These 6 probability values are then sorted to determine the category of the ECG signal.

Our framework is built using TensorFlow and executed on an NVIDIA Tesla V100 GPU. In our proposed CNN structure, the kernel size of primary convolution is set to (1, 5), while the max-pooling layer is fixed to 2, with the pooling stride fixed to 2. For efficient computation, we utilize an optimization method called mom. During training, we employ a weighted loss function known as cross-entropy loss. The hyperparameters for our model are configured as follows: a batch size of 100, a learning rate of 0.00008, and 500 training epochs. To evaluate the model’s performance on the test dataset, we calculated metrics such as accuracy, sensitivity, specificity, F1 score, and AUC.

**Fig. 2.**
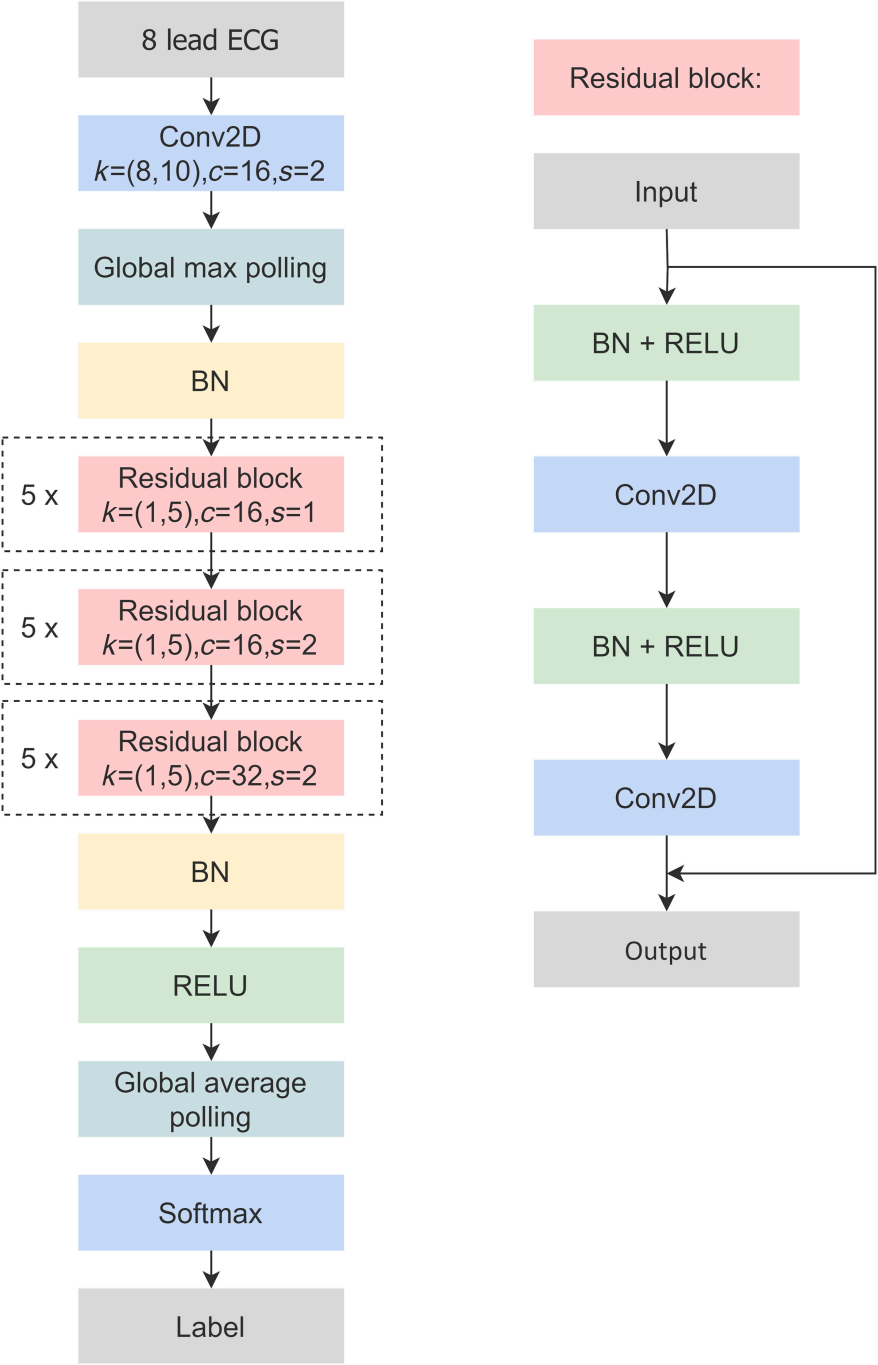
Diagnosis deep learning model with residual blocks.

Since all the 5 types of abnormal ECG are characterized by wide QRS waves, we first adopted QRS detection algorithms such as PT algorithm [16] to determine the R point and segmented the original ECG data to obtain the heartbeat data as a separate heartbeat dataset. The original ECG dataset and heartbeat dataset were trained using the above model framework, and the first 6 classification model and the second 6 classification model were obtained. Then, XGBoost was used to train the ECG data through 2 sets of feature vectors obtained by two 6-classification models, and the final fusion 6-classification model was obtained. XGBoost is a scalable machine learning system for tree boosting. The discrimination of the 2 types of data in WPW is more difficult, and the data size is smaller. Because the main morphological difference between WPW-A and WPW-B is reflected in channel V1, channel V1 data are used to train the dichotomous classification model, which can further improve the recognition rate of these 2 classes. Finally, the fusion 6 classification model and the 2 classification models are combined and trained by XGBoost to get the final model. This method can make the overall model more accurately capture the important features of the 5 categories of diseases, improve the prediction ability of the model, enhance the detection rate of the 5 categories of diseases, and reduce the risk (Fig. 2).

### Statistical analysis

The accuracy, AUROC, specificity, sensitivity, and F1 score, with 2-sided 95% confidence intervals (CIs), were used to assess the performance of the model. Confusion matrices were assessed to evaluate whether predictions were discordant with the cardiologists’ diagnosing as previously described [7]. Statistical analysis of the data was performed by using one-way analysis of variance (ANOVA), followed by post hoc analysis with the SPSS software (version 26). *P* < 0.05 was considered to indicate a statistically significant difference.

## Results

**Fig. 3.**
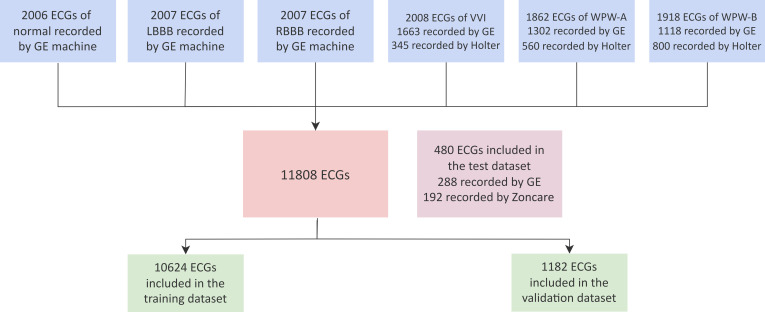
Profiles of the dataset ECGs in the training and validation dataset were recorded from 2012 January 1 to 2020 November 30. The ECGs in the test dataset were recorded between 2014 January 1 and 2017 November 30. To prevent time series dependencies of the datasets, we ensured that data from any individual patient were included in the training, validation, or testing set only. Each patient in the test dataset only contributed one ECG to that set. GE machine, GE-Marquette type 3500 or 5500 ECG machine.

Our dataset includes 11,808 10-s, 12-channel ECGs from 8,542 patients between 2012 January 1 and 2020 October 30. The average number of ECGs in each group was 1,968, and 61.1% were men. The training (10,624 ECGs) and validation datasets (1,182 ECGs) were divided in the ratio of 9:1. Our deep learning model was then validated using the test dataset comprising 480 ECGs recorded between 2014 January 1 and 2017 November 30 by GE machines, corresponding to 480 unique patients [264 (54.9%) men; mean age, 54.7 years (SD 15.8)] (Fig. 3 and Table S1). Further, the performance of the model was compared to the diagnoses of 6 ECG physicians in cardiology departments of Tongji Hospital or The First People’s Hospital of Jingmen with an average age of 29.7 ± 1.86 years and a working experience of 3.25 ± 1.63 years (Table S2).

More detailed information on the ECG number, mean age, and male ratio in the training and testing datasets is given in Table S1. In the training dataset, the patients in the WPW-A, WPW-B, and normal group were much younger than those in the LBBB, RBBB, and AVPR group. Each of these 5 arrhythmias incorporated more male than female patients. The number of samples for each abnormality varies between 1,862 (for WPW-A) and 2008 (for RBBB), consisting of a well-balanced dataset. In normal, LBBB, and RBBB groups, all the ECG data were recorded by GE machines, whereas 17.2%, 30.1%, and 41.7% of ECGs in the AVPR, WPW-A, and WPW-B group, respectively, were recorded by Holter machines (Fig. 3). The patients in the TJ-Test set shows consistent distribution in each group compared to that in the training dataset.

The model was assessed with 4-channel (I, II, V_1_, and V_5_) and 8-channel (I, II, and V_1_ to V_6_) formats, compared to the 12-channel format (I, II, III, aVR, aVL, aVF, and V_1_ to V_6_), in assessing the 6 abnormalities. There was no significant difference between these 3 groups using one-way ANOVA analysis (*P* > 0.05). The 8-channel format exhibited a higher average F1 score of 0.969 (0.943 to 0.997) and a mean AUC score of 0.997 (0.975 to 1.00) than did the 4-channel format with an average F1 score of 0.952 (0.931 to 0.969) and an AUC score of 0.996 (0.976 to 1.00), and the 12-channel format with an average F1 of score 0.95 (0.927 to 0.97) and an AUC score of 0.994 (0.971 to 1.00). The 8-channel format also demonstrated a numerically slightly higher but not statistically significant average sensitivity and specificity than the 4- and 12-channel formats. When comparing the ROC curves and confusion matrix, the 3 channel formats showed similar distribution tendency in CNN performance (Figs. S1 and S2).

The deep learning model on the 8-channel format showed significantly higher performance than that of physicians’ (*P* < 0.05). It achieved an accuracy of 456 (95.0%) out of 480 cases in the test dataset, surpassing that of physicians’ evaluations with an accuracy of 432 (89.9%) out of 480 cases. The model’s mean F1 score for 6 abnormalities was 0.969 (0.943 to 0.997), which exceeded the mean score 0.898 (0.863 to 0.932) of the physicians. Furthermore, the mean AUC score of our algorithm on the 8-channel format for diagnosing these 6 classes is 0.997 (0.975 to 1.00), which is higher than the physicians’ AUC score of 0.941 (0.918 to 0.963). The model’s AUC scores for each of the 6 abnormalities were also higher than the average scores of the 6 physicians (Table 1). The model performed with an average sensitivity of 0.969 (0.94 to 0.995) and specificity of 0.994 (0.988 to 1.00), both of which were significantly higher than those of the physicians’ evaluations, which yielded a sensitivity of 0.902 (0.858 to 0.946) and a specificity of 0.980 (0.97 to 0.99) (Table 1 and Table S3).

**Table 1.** Performance of the deep learning model with the 8-channel format in abnormality diagnosis compared to that of the physicians

	8-Channel model on TJ-Test set				Physicians on TJ-Test set				8-Channel model on JX-Test set			
	AUC (95% CI)	Sensitivity (95% CI)	Specificity (95% CI)	F1 (95% CI)	AUC (95% CI)	Sensitivity (95% CI)	Specificity (95% CI)	F1 (95% CI)	AUC (95% CI)	Sensitivity (95% CI)	Specificity (95% CI)	F1 score (95% CI)
Normal	0.998 (0.98–1.00)	0.943 (0.923–0.962)	0.998 (0.979–1.00)	0.964 (0.94–0.988)	0.978 (0.969–0.987)	0.983 (0.975–0.991)	0.973 (0.958–0.988)	0.92 (0.882–0.958)	0.999 (0.976–1.00)	0.955 (0.93–0.981)	0.992 (0.972–1.00)	0.955 (0.928–0.982)
LBBB	1.00 (0.972–1.00)	0.989 (0.961–1.00)	0.995 (0.99–1.00)	0.983 (0.958–0.999)	0.959 (0.929–0.989)	0.925 (0.864–0.987)	0.993 (0.99–0.996)	0.944 (0.911–0.976)	0.998 (0.971–1.00)	0.981 (0.958–1.00)	0.983 (0.963–0.999)	0.953 (0.928–0.979)
RBBB	0.999 (0.975–1.00)	0.988 (0.954–0.995)	0.995 (0.99–1.00)	0.982 (0.955–0.997)	0.959 (0.934–0.984)	0.925 (0.873–0.977)	0.994 (0.99–0.998)	0.945 (0.921–0.969)	0.998 (0.972–1.00)	1.00 (0.999–1.00)	0.984 (0.961–1.00)	0.957 (0.932–0.982)
AVPR	1.00 (0.981–1.00)	0.987 (0.965–1.00)	0.995 (0.989–1.00)	0.981 (0.958–997)	0.966 (0.934–0.981)	0.935 (0.907–0.963)	0.998 (0.996–0.999)	0.96 (0.943–0.976)	0.989 (0.966–1.00)	0.955 (0.926–0.984)	0.992 (0.977–1.00)	0.955 (0.935–0.975)
WPW-A	0.998 (0.974–1.00)	0.962 (0.934–0.989)	0.988 (0.98–0.993)	0.95 (0.91–0.989)	0.957 (0.952–0.97)	0.975 (0.954–0.996)	0.938 (0.917–0.96)	0.855 (0.813–0.898)	0.972 (0.949–0.995)	0.896 (0.876–0.916)	0.963 (0.94–0.986)	0.86 (0.835–0.885)
WPW-B	0.989 (0.97–1.00)	0.94 (0.897–0.984)	0.992 (0.987–0.997)	0.951 (0.913–0.989)	0.826 (0.783–0.87)	0.669 (0.574–0.764)	0.984 (0.968–1.00)	0.762 (0.708–0.816)	0.946 (0.923–0.969)	0.746 (0.726–0.766)	0.983 (0.96–0.999)	0.822 (0.797–0.847)
Average	0.997 (0.975–1.00)	0.969 (0.94–0.995)	0.994 (0.988–1.00)	0.969 (0.943–0.997)	0.941 (0.918–0.963)	0.902 (0.858–0.946)	0.98 (0.97–0.99)	0.898 (0.863–0.932)	0.984 (0.975–1.00)	0.922 (0.94–0.995)	0.983 (0.988–1.00)	0.917 (0.943–0.997)

**Fig. 4.**
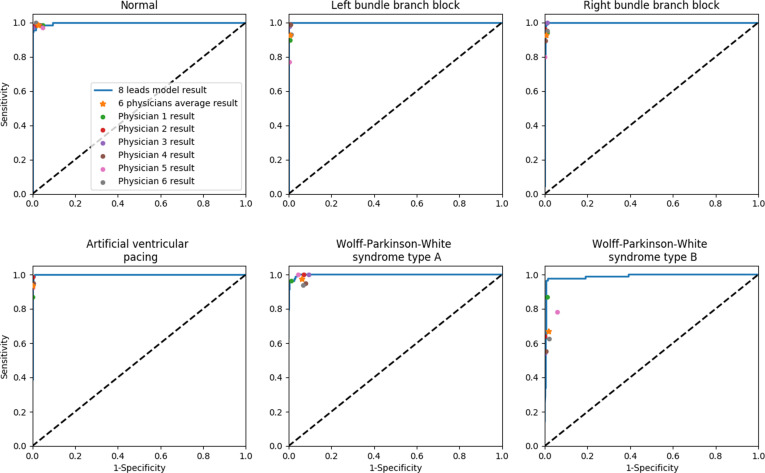
For 6 abnormalities, ROC curves of the prediction sensitivity of the deep learning model with the 8-channel format compared to that for 6 physicians. Individual physician performance is indicated by the dots, and average physician performance is indicated by the star.

**Fig. 5.**
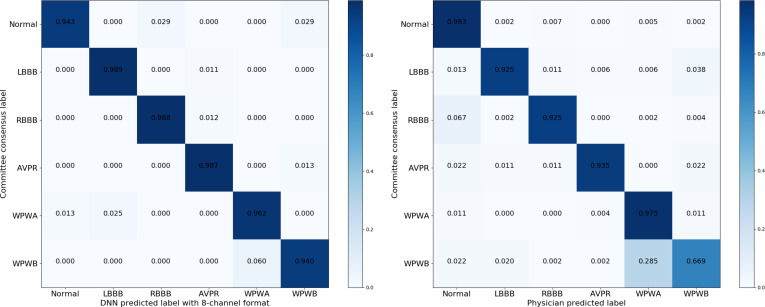
Confusion matrices for the deep learning model with the 8-channel format as compared to physician performance.

When comparing the ROC curves of the model with those of the 6 physicians, the model performed better (Fig. 4). The confusion matrices of the model (Fig. 5) revealed similar distribution tendency in CNN performance and physician performance. These results indicate that our AI-enabled model outperforms physician assessment for most of the 6 abnormalities.

For the JX-Test set, our model on the 8-channel format had a mean F1 score of 0.917 (0.943 to 0.997) and ROC of 0.984 (0.975 to 1.00) in classifying these 6 abnormalities, exhibiting a similar effect compared to the TJ-Test set (Table 1).

For the external validation data [13], our model had a mean F1 score of 0.708 for LBBB and 0.828 for RBBB with 8-channel format data, which were notably lower than those in the test dataset from Tongji Hospital (0.983 for LBBB and 0.982 for RBBB) (Table S4). This decline in performance may be attributed to the limited number of ECGs in the public dataset, leading to less stable estimates. Moreover, differences in patient demographics, ECG acquisition protocols, and labeling criteria between datasets may have contributed to reduced generalizability. These findings underscore the need for further validation on larger and more diverse external datasets to better assess the robustness of the model.

## Discussion

In our study, we developed an AI-based 8-channel ECG diagnosis model using a large cohort dataset encompassing normal ECGs and 5 challenging abnormalities featuring a wide QRS complex in sinus rhythm and an artificial ventricular pacing rhythm. In order to objectively demonstrate the capability of our model, the dataset was constructed with balanced and diversity components, with ECG data in standard 12-channel type collected from diverse instruments including convolutional GE machines, Zoncare machine, and dynamic ECG machines. The patients involved were composed of different genders and age ranges.

While the 12-channel ECG is the clinical standard, it is often difficult to collect all 12 channels in some real-world settings. Recent studies have shown that reduced-channel systems, such as 6 to 8 channels, can still achieve reliable performance in detecting rhythm and structural abnormalities. For example, using channels I, II, and V1 to V6, an 8-channel setup showed an average AUC only 0.3% lower than that of a full 12-channel system, suggesting its practicality for clinical use [17].

In clinical practice, accurate ECG diagnosis is paramount for timely treatments [18]. Valuable information such as the presence and severity of arrhythmias can be provided by the regular surface ECGs. However, the precise diagnosis of rhythm or conduction abnormalities characterized by a wide QRS complex can be challenging and may result in erroneous diagnoses, especially for those doctors with less clinical experience [18]. The WPW syndrome is mainly characterized by a prolonged QRS duration with initial slurring of QRS upstroke (delta wave) and a short PR interval [19]. Therefore, AVPR, which displays a short PR interval like pre-excitation and may be misleading, was contained in our datasets. The WPW syndrome was described as a “functional bundle branch block” because of their similar ECG waveforms [20]. Both the BBB and the pre-excitation syndrome display a prolonged QRS duration (>0.12 s). More specifically, WPW-A exhibits positive delta waves and QRS waves in all precordial channels. These features are often confused with RBBB and other diseases such as right ventricular hypertrophy (RVH) or posterior wall MI. Likewise, WPW-B displays negative QRS waves in channel V1 and positive delta and QRS waves in V4 to V6, which is often misdiagnosed as LBBB, left ventricular hypertrophy (LVH), and anterior/septal wall MI. The length of the PR interval is a crucial factor to distinguish WPW syndromes from these similar abnormalities [19]. However, these characteristics may not be displayed simultaneously in one ECG, which enormously increases the difficulty for accurate diagnosis in clinical practice. Therefore, developing an AI-based model that can efficiently differentiate between these highly similar ECG waveforms is crucial, as it will facilitate precise diagnosis and appropriate treatment of patients.

Thus, as we know, we are the first to investigate the simplified channel formats with 3 different formats to detect the wide QRS complex. In the normal condition, the 12-channel ECGs with 10 electrodes applied are utilized most widely. According to our study, we found that 8- and 4-channel formats showed comparable or even better performance than did the 12-channel format, where the 8-channel format stands out with a relatively high average F1 scores. For classifying WPW-A and RBBB, WPW-B and LBBB, and AVPR, the 3 channel formats showed comparable performance, with an average F1 score, specificity, and AUC higher than 0.9. The 4-channel format shows varied sensitivity in classifying RBBB [0.88 (0.86 to 0.899)] and LBBB [1.00 (0.98 to 1.00)]. Thus, the limited channel format with only channels I, II, V_1_, and V_5_is consistent to the necessary channels for the differentiation of WPW-A, WPW-B, RBBB, and LBBB cases [21]. This is a convenient way in the future to implement data storage, processing, and application.

In addition, the 3 channel formats show relatively high performance in distinguishing AVPR from the normal group and other wide QRS complex abnormalities, with a sensitivity of 1 (0.98 to 1.00) in the 4-channel format and an AUC of 1.00 (0.981 to 1.00) in the 8-channel format. AVPR showed obvious pacemaker signals in the ECG and also confused a wide QRS complex compared to other abnormalities. Thus, our model showed superior performance in collecting the specific characteristics in classifying AVPR.

As for the 8-channel format, we compared it to that of the physicians’ result. It showed that the 8-channel format exhibited superior performance in detecting the 5 abnormalities with a wide QRS complex, even in distinguishing WPW-A from RBBB, and WPW-B from LBBB. In the physicians’ level, it showed a reduced F1 score in diagnosing WPW-A [0.855 (0.813 to 0.898)] and WPW-B [0.762 (0.708 to 0.816)]. We consider that it is not necessary to detect WPW into type A and type B; thus, many physicians have forgotten the detailed features of these 2 types, making an inferior performance in classifying them. Our model demonstrated superior precision and F1 scores when compared to the analysis of 6 physicians, indicating its proficiency in extracting and differentiating intricate features even among highly similar ECG waveforms. We also found that the false negative (FN) and false positive (FP) rates remained low in our model for diagnosing these 6 classes. The model showed a relatively comparable performance in a multicenter hospital and the external published dataset. These findings underscore our model’s potential as a promising clinical tool in AI-based ECG diagnosis, particularly in accurately distinguishing normal ECGs, LBBB, RBBB, AVPR, and 2 types of WPWs (WPW-A and WPW-B) characterized by a wide QRS complex. This further advances the field of AI-assisted identification of wide QRS complex abnormalities. However, F1 scores for LBBB and RBBB declined notably in the public dataset, which may be related to the limited number of ECGs. Smaller sample sizes can lead to less stable performance. Additionally, differences in patient characteristics, ECG acquisition, or labeling criteria between datasets may influence generalizability. These findings highlight the importance of validating the model on broader, more diverse external data.

To put our findings in context, Zhu et al. [7] reported an F1 score of 0.887 and an AUC of 0.983 using full 12-channel ECGs for multiple heart abnormalities. Makimoto et al. [8] achieved about 81% accuracy and 83% F1 score detecting MI with a CNN. Senoner et al. [9] developed an AI model to locate accessory pathways in WPW syndrome, achieving 85.7% accuracy. In contrast, our 8-channel model reached higher scores, with an average F1 score of 0.969 and AUC of 0.997 for wide QRS abnormalities. Luo et al. [12] used an 8-channel ECG to detect atrial septal defects, with an F1 score of 0.81 and AUC of 0.87, lower than our results. This suggests that a simplified channel setup combined with AI can deliver accurate diagnoses efficiently. Besides, cardiac auscultation is another important methods for detecting heart diseases. Zhao et al. [22] have analyzed the auscultatory characteristics in diagnosing various heart diseases using a deep learning model, which indicates that we can combine ECG with auscultation signals to construct the diagnostic model.

It is worth noting that our study focused on the differential diagnosis of morphologically similar ECG abnormalities. More specifically, WPW-A is often confused with RBBB and other diseases such as RVH or posterior wall MI. Likewise, WPW-B is often misdiagnosed as LBBB, left ventricular hypertrophy, and anterior/septal wall MI. The length of the PR interval is a crucial factor to distinguish WPW syndromes from these similar abnormalities. Pacemaker ECGs can also be confused with RBBB. Due to the limited amount of data on WCT, including ventricular tachycardia and supraventricular tachycardia with aberrancy, we need more time to collect sufficient meaningful data for analysis. We plan to conduct research on WCT in future studies. Although our study included the most classical wide QRS complex abnormalities, other abnormalities like WCT were not included, nor the sinoatrial conduction caused by hyperkalemia or the prolongation of QRS wave caused by nonspecific intraventricular block. ECG changes similar to the WPW syndrome such as ventricular hypertrophy and MI were not included. The exclusion of wide WCT from this study necessarily limits its immediate clinical applicability to acute arrhythmia differentiation. Future work is essential to develop complementary models or integrated systems capable of reliably classifying tachycardias at high rates, a vital requirement for emergency and acute cardiac care. Additionally, it lacks interpretability analysis such as heatmaps to reveal the mechanism of our model in identifying these 6 kinds of abnormalities, which we will research in the next study. Interpretability is crucial in clinical AI applications as it helps build physician trust, facilitates understanding of model decisions and failures, and supports safer implementation in practice, which is important in this study.

## Conclusion

Our model distinguishes a range of distinct abnormalities focusing on abnormal morphology on QRS complexes in normal heartbeats with high accuracy, providing a foundation for artificial intelligence (AI)-aided clinical decision-support systems in ECG differential diagnosis.

## Ethical Approval

This study was approved by the ethics committee of Tongji Hospital of Huazhong University of Science and Technology (approval number TJ-IRB202507044).

## Data Availability

The datasets generated and analyzed during the current study are available from the corresponding author on reasonable request.
