# Supplementary material for: Artificial Intelligence-Enabled 8-Channel ECG Diagnosing of Abnormalities with Wide QRS Complexes
Source: Health Data Sci. 2026 Feb 5;6:0265. doi: 10.34133/hds.0265 (PMC12873062; doi:10.34133/hds.0265)

**Supplementary Information**

Supplement to: Artificial intelligence-enabled identification of ECG abnormalities with wide QRS complexes: a cohort study

**Supplementary Table 1: Mean age and the standard deviation (SD) of each rhythm class.** The average age was calculated by accepting all the recorded ECG samples in our dataset, without considering the patient repetition for this statistical calculation.

|  | Training dataset | | | Testing dataset | | |
| --- | --- | --- | --- | --- | --- | --- |
|  | No | age | Male ratio | No | age | Male ratio |
| **Normal** | 2006 | 43.6  (±11.9) | 41.0% | 70 | 48.7  (±17.3) | 54.3% |
| **Left bundle branch block** | 2007 | 66.1  (±13.7) | 57.4% | 87 | 69.7  (±11.8) | 50.6% |
| **Right bundle branch block** | 2007 | 57.4  (±16.5) | 71.0% | 84 | 53.6  (±20.9) | 53.6% |
| **Artificial ventricular pacing** | 2008 | 69.6  (±15.1) | 77.3% | 77 | 73.9  (±13.0) | 55.8% |
| **Wolff-Parkinson-White syndrome type A** | 1862 | 42.4  (±15.2) | 63.6% | 79 | 44.8  (±14.7) | 59.5% |
| **Wolff-Parkinson-White syndrome type B** | 1918 | 43.4  (±14.1) | 56.1% | 83 | 37.5  (±17.0) | 55.4% |
| **Average** | 1968 | 53.8  (±14.5) | 61.1% | 80 | 54.7  (±15.8) | 54.9% |

**Supplementary Table 2: Information of 6 Physicians**.

| Number | Mean of working year (SD) | Mean age | Female | Male |
| --- | --- | --- | --- | --- |
| 6 | 3.25±1.63 | 29.67±1.86 | 4 | 2 |

**Supplementary Table 3: Performance of the deep learning model with 4-, 8- and 12-channel format in abnormalities diagnosis compared to that of physicians’ (Table 3A) and the significant analysis using one-way ANOVA (Table 3B)**

| **A** | **4-channel Model** | | | | **8-channel Model** | | | | **12-channel Model** | | | | **Physicians** | | | |
| --- | --- | --- | --- | --- | --- | --- | --- | --- | --- | --- | --- | --- | --- | --- | --- | --- |
|  | **AUC (95% CI)** | **sensitivity (95% CI)** | **specificity (95% CI)** | **F1 score (95% CI)** | **AUC (95% CI)** | **sensitivity (95% CI)** | **specificity (95% CI)** | **F1 score (95% CI)** | **AUC (95% CI)** | **sensitivity (95% CI)** | **specificity (95% CI)** | **F1 score (95% CI)** | **AUC (95% CI)** | **sensitivity (95% CI)** | **specificity (95% CI)** | **F1 score (95% CI)** |
| **Normal** | 0.998 (0.978-1.00) | 0.929 (0.909-0.95) | 0.985 (0.965-1.00) | 0.922 (0.902-0.942) | 0.998 (0.98-1.00) | 0.943 (0.923-0.962) | 0.998 (0.979-1.00) | 0.964 (0.94-0.988) | 0.998 (0.975-1.00) | 0.914 (0.894-0.934) | 0.988 (0.965-1.00) | 0.921 (0.899-0.943) | 0.978 (0.969-0.987) | 0.983 (0.975-0.991) | 0.973 (0.958-0.988) | 0.92 (0.882-0.958) |
| **LBBB** | 0.999 (0.979-1.00) | 1.00 (0.98-1.00) | 0.995 (0.975-1.00) | 0.989 (0.969-1.00) | 1.00 (0.972-1.00) | 0.989 (0.961-1.00) | 0.995 (0.99-1.00) | 0.983 (0.958-0.999) | 0.999 (0.976-1.00) | 1 (0.98-1.00) | 0.992 (0.969-1.00) | 0.983 (0.961-0.997) | 0.959 (0.929-0.989) | 0.925 (0.864-0.987) | 0.993 (0.99-0.996) | 0.944 (0.911-0.976) |
| **RBBB** | 0.99 (0.97-1.00) | 0.88 (0.86-0.899) | 0.995 (0.975-1.00) | 0.925 (0.905-0.945) | 0.999 (0.975-1.00) | 0.988 (0.954-0.995) | 0.995 (0.99-1.00) | 0.982 (0.955-0.997) | 0.985 (0.962-1.00) | 0.892 (0.872-0.912) | 0.99 (0.967-1.00) | 0.92 (0.898-0.942) | 0.959 (0.934-0.984) | 0.925 (0.873-0.977) | 0.994 (0.99-0.998) | 0.945 (0.921-0.969) |
| **AVPR** | 0.998 (0.978-1.00) | 1 (0.98-1.00) | 0.99 (0.97-1.00) | 0.975 (0.955-0.995) | 1.00 (0.981-1.00) | 0.987 (0.965-1.00) | 0.995 (0.989-1.00) | 0.981 (0.958-997) | 0.998 (0.977-1.00) | 0.987 (0.967-0.999) | 0.99 (0.968-1.00) | 0.968 (0.946-0.99) | 0.966 (0.934-0.981) | 0.935 (0.907-0.963) | 0.998 (0.996-0.999) | 0.96 (0.943-0.976) |
| **WPW-A** | 0.999 (0.979-1.00) | 0.987 (0.967-1.00) | 0.985 (0.965-1.00) | 0.957 (0.937-0.977) | 0.998 (0.974-1.00) | 0.962 (0.934-0.989) | 0.988 (0.98-0.993) | 0.95 (0.91-0.989) | 0.998 (0.975-1.00) | 0.975 (0.955-0.995) | 0.99 (0.97-1.00) | 0.963 (0.941-0.985) | 0.957 (0.952-0.97) | 0.975 (0.954-0.996) | 0.938 (0.917-0.96) | 0.855 (0.813-0.898) |
| **WPW-B** | 0.989 (0.969-0.99) | 0.916 (0.896-0.936) | 0.992 (0.972-1.00) | 0.938 (0.918-0.958) | 0.989 (0.97-1.00) | 0.94 (0.897-0.984) | 0.992 (0.987-0.997) | 0.951 (0.913-0.989) | 0.986 (0.963-0.998) | 0.928 (0.908-0.948) | 0.99 (0.968-1.00) | 0.939 (0.917-0.961) | 0.826 (0.783-0.87) | 0.669 (0.574-0.764) | 0.984 (0.968-1.00) | 0.762 (0.708-0.816) |
| **Average** | 0.996 (0.976-1.00) | 0.952 (0.932-0.964) | 0.99 (0.971-1.00) | 0.952 (0.931-0.969) | 0.997 (0.975-1.00)* | 0.969 (0.94-0.995) | 0.994 (0.988-1.00) | 0.969 (0.943-0.997) | 0.994 (0.971-1.00) | 0.95 (0.93-0.965) | 0.99 (0.968-1.00) | 0.95 (0.927-0.97) | 0.941 (0.918-0.963) | 0.902 (0.858-0.946) | 0.98 (0.97-0.99) | 0.898 (0.863-0.932) |

| B | **AUC** | **Sensitivity** | **Specificity** | **F1** |
| --- | --- | --- | --- | --- |
| **P_A_** | 0.254 | 0.432 | 0.113 | 0.086 |
| **P_B_** | 0.010 | 0.019 | 0.001 | 0.002 |

*P_A_ refers to the statistical P-value between the 8-channel ECG model and the 4/12-channel ECG models. P_B_ refers to the statistical P-value between physicians and the 8-channel ECG model.*

**Supplementary Figure 1. ROC curves of** *the prediction sensitivity of the deep learning model with 4-, 8-and 12-channel compared to that for 6 physicians. Individual physician performance is indicated by the dots and averaged physician performance is indicated by the star.*


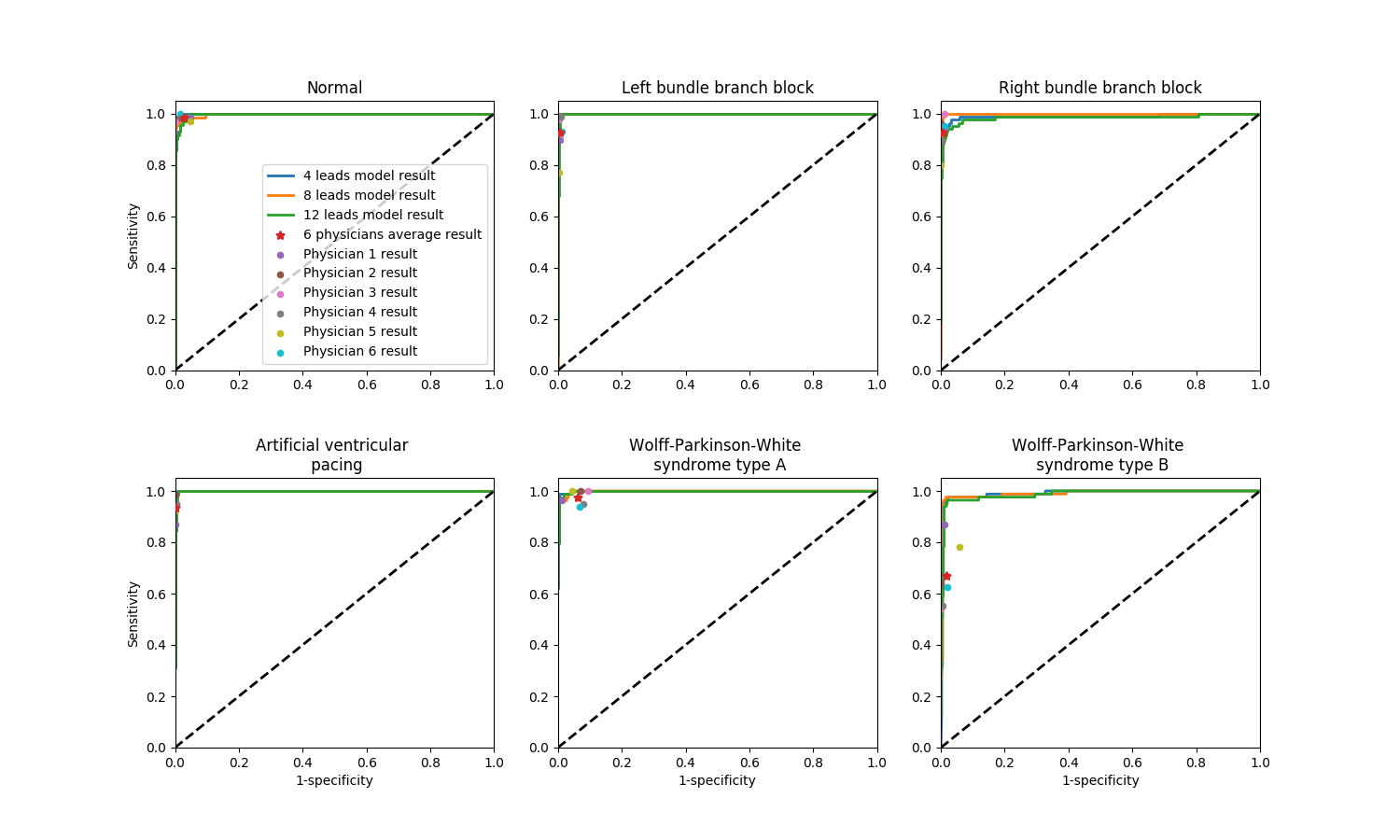


**Supplementary Figure 2. Confusion matrices for the deep learning model with 4-, 8- and 12-channel format as compared to physician performance**

**
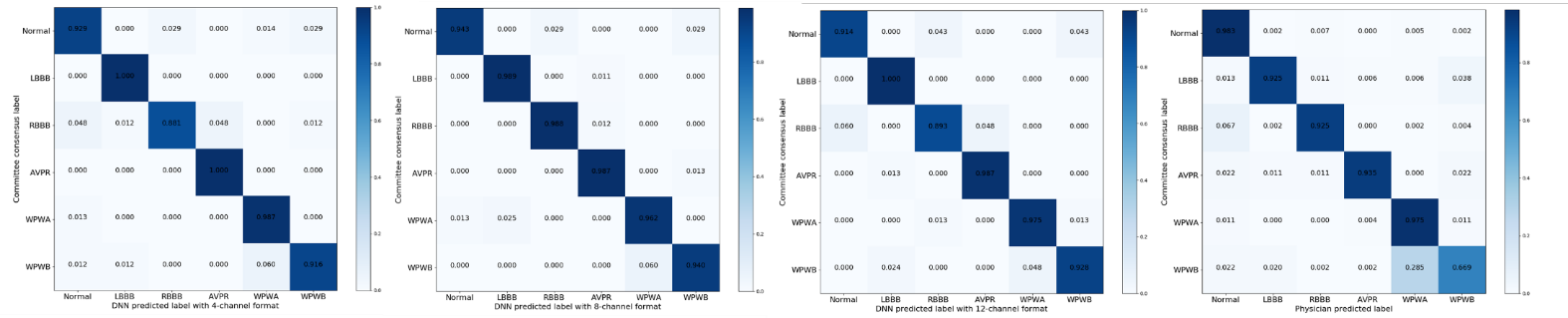
**

**Supplementary Figure 3. ROC curves of the deep learning model with 8-channel format in abnormalities diagnosis in** **JX-Test set.**


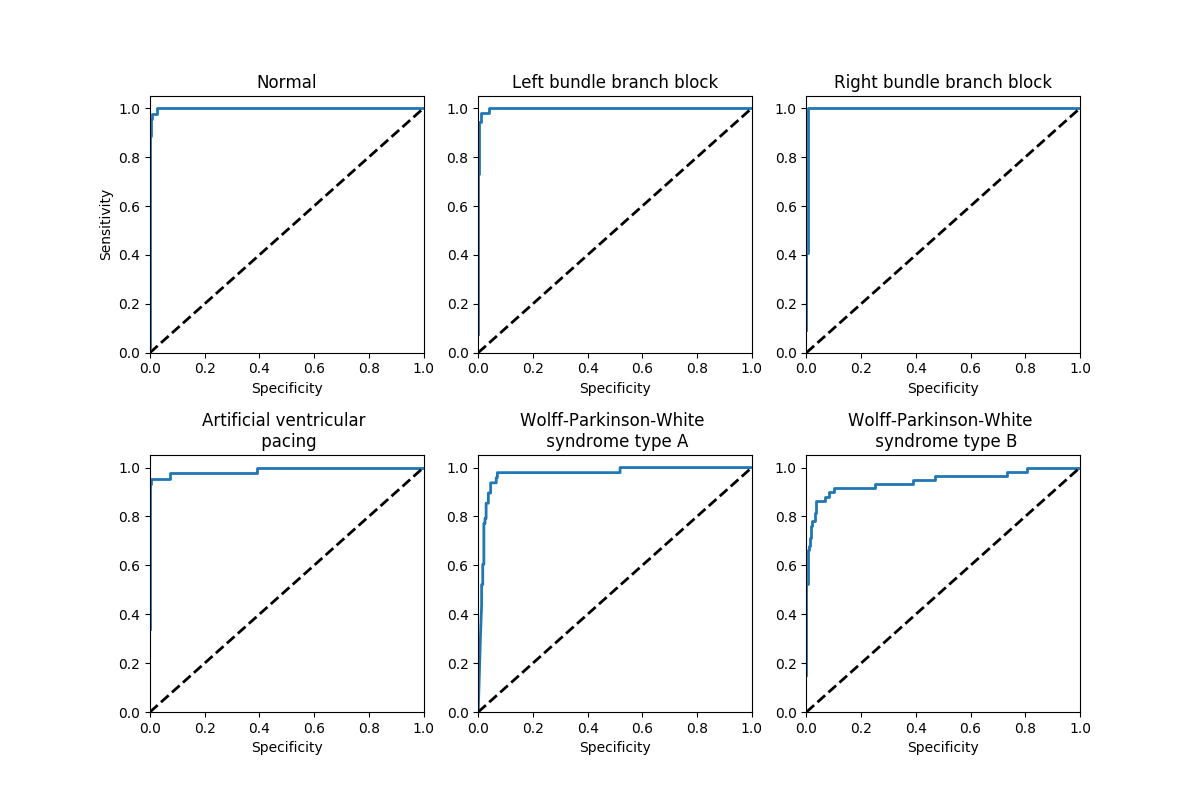


**Supplementary Figure 4. Confusion matrix of the deep learning model with 8-channel format in abnormalities diagnosis in** **JX-Test set.**


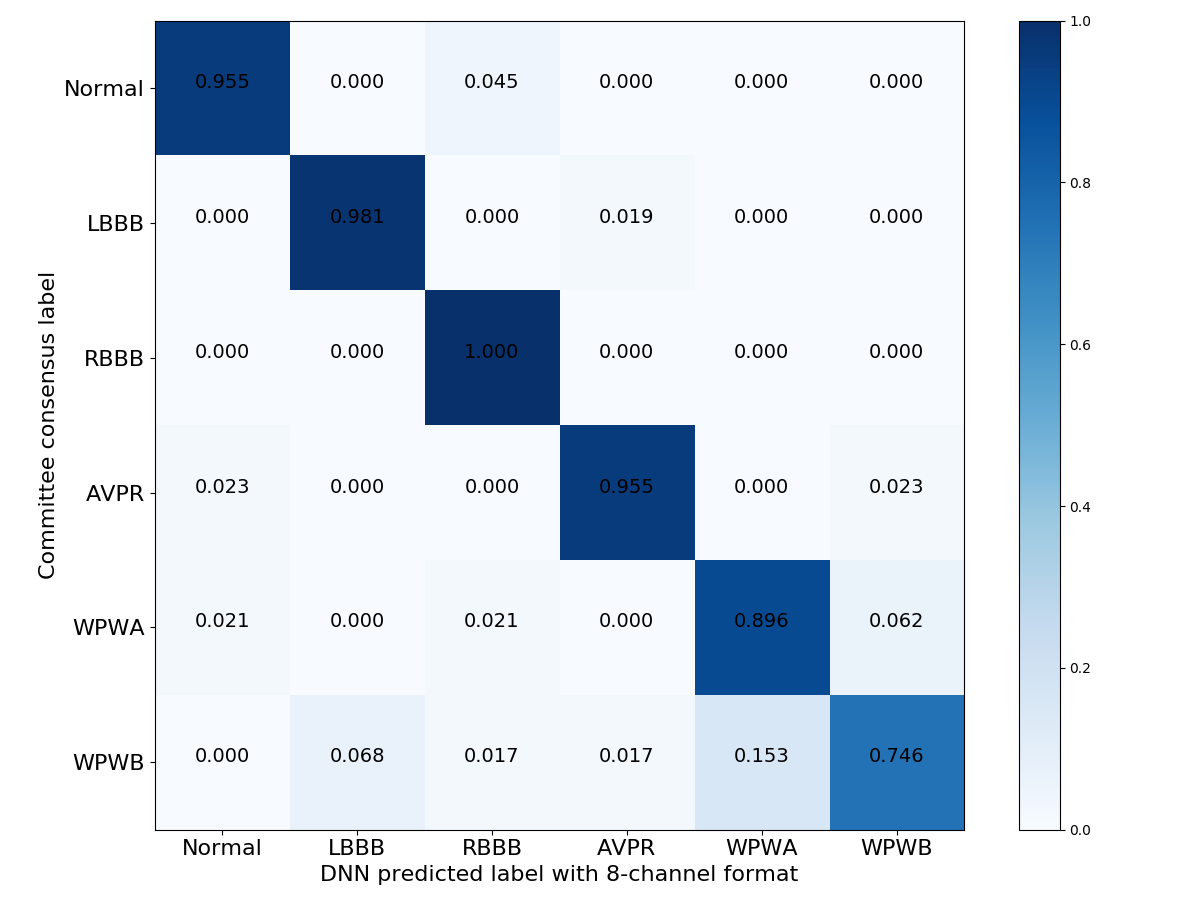


**Supplementary table 4. Performance of the deep learning model with 8-channel format in abnormalities diagnosis in external database.**

|  | **Precision** | **AUC** | **Sensitivity** | **Specificity** | **F1** |
| --- | --- | --- | --- | --- | --- |
| **LBBB** | 0.944 | 0.8676 | 0.5667 | 0.9706 | 0.7083 |
| **RBBB** | 1 | 0.9127 | 0.7059 | 1 | 0.8276 |

**Supplementary Figure 5. ROC curves of the deep learning model with 8-channel format in abnormalities diagnosis in external databse.**


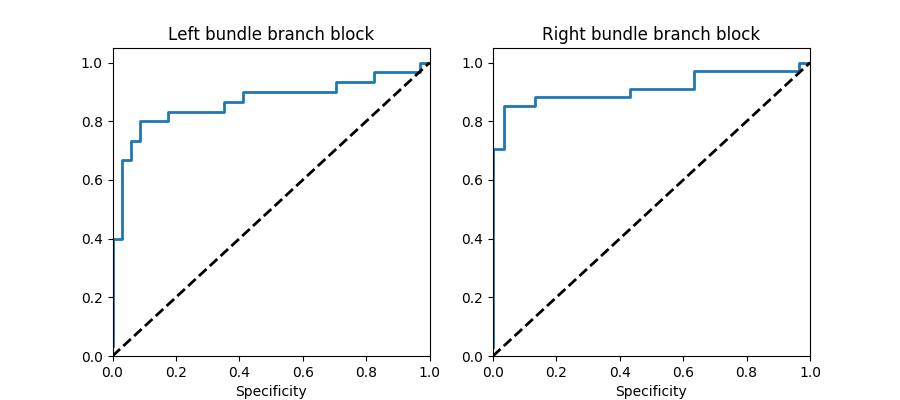


**Supplementary Figure 6. Confusion matrix of the deep learning model with 8-channel format in abnormalities diagnosis in external database.**


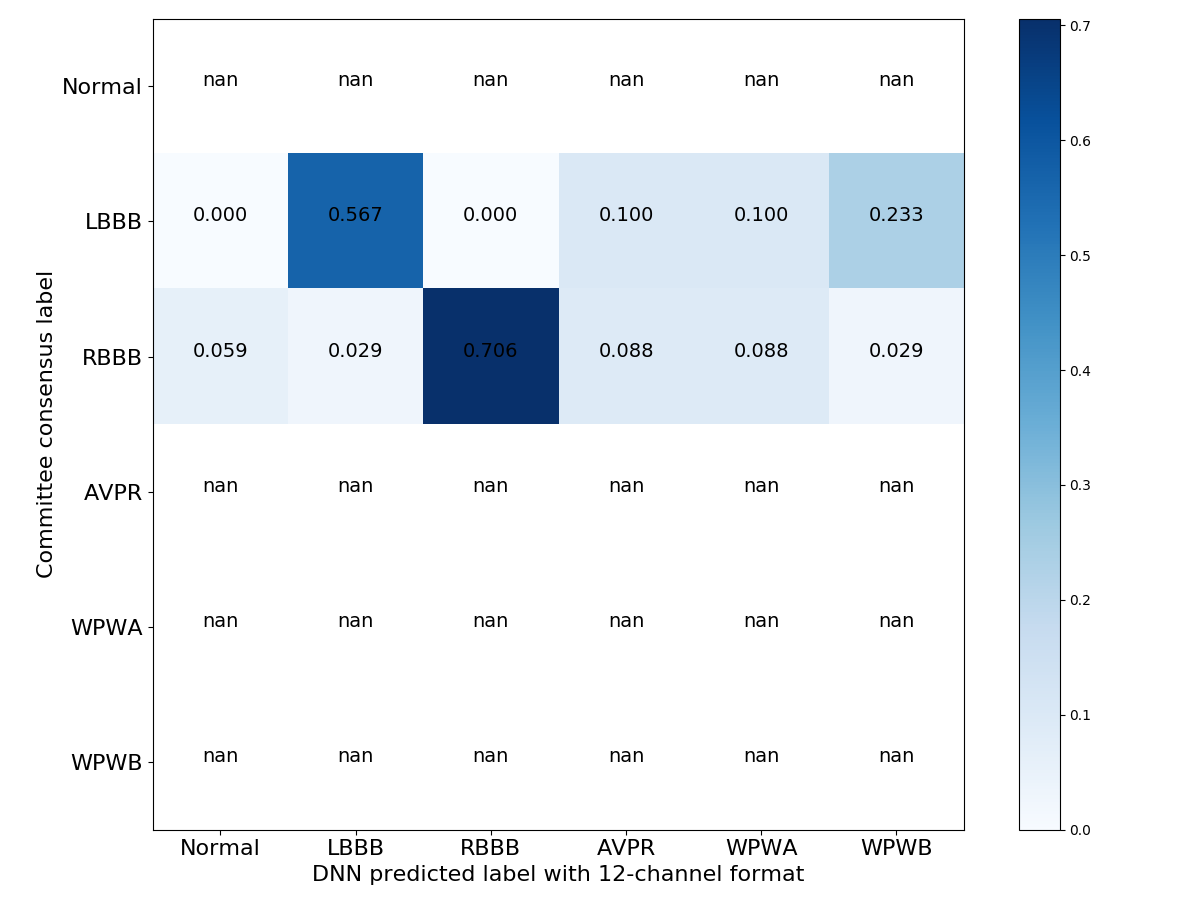

Supplement: Supplementary 1 — Figs. S1 to S6 Tables S1 to S4 [file hds.0265.f1.zip › renamed_6ebbc.docx]
