# Supplementary figures and images for: Artificial Intelligence-Enabled 8-Channel ECG Diagnosing of Abnormalities with Wide QRS Complexes
Source: Health Data Sci. 2026 Feb 5;6:0265. doi: 10.34133/hds.0265 (PMC12873062; doi:10.34133/hds.0265)

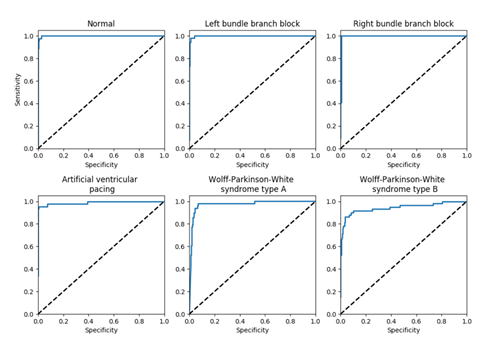

Supplement: Supplementary 1 — Figs. S1 to S6 Tables S1 to S4 [file hds.0265.f1.zip › Supplementary Figure 3. ROC of JX-Test set with 8-channel.png]

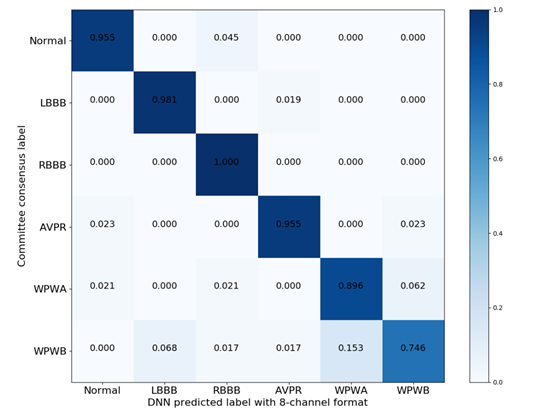

Supplement: Supplementary 1 — Figs. S1 to S6 Tables S1 to S4 [file hds.0265.f1.zip › Supplementary Figure 4. JX-Test set Confusion matrix.png]
